# Can Consumers Trust Web-Based Information About Celiac Disease? Accuracy, Comprehensiveness, Transparency, and Readability of Information on the Internet

**DOI:** 10.2196/ijmr.2010

**Published:** 2012-04-04

**Authors:** Shawna L McNally, Michael C Donohue, Kimberly P Newton, Sandra P Ogletree, Kristen K Conner, Sarah E Ingegneri, Martin F Kagnoff

**Affiliations:** 1 Wm. K. Warren Medical Research Center for Celiac DiseaseDepartment of MedicineUniversity of California, San DiegoLa Jolla, CAUnited States; 2 Division of Biostatistics and BioinformaticsDepartment of Family and Preventive MedicineUniversity of California, San DiegoLa Jolla, CAUnited States; 3 Division of GastroenterologyDepartment of PediatricsUniversity of California, San DiegoLa Jolla, CAUnited States; 4 School of MedicineUniversity of California, San DiegoLa Jolla, CAUnited States; 5 Point Loma Nazarene UniversitySan Diego, CAUnited States; 6 University of ArizonaDepartment of Nutritional SciencesTucson, AZUnited States

**Keywords:** Celiac disease, health information, website accuracy, website comprehensiveness, website transparency, website quality

## Abstract

**Background:**

Celiac disease is an autoimmune disease that affects approximately 1% of the US population. Disease is characterized by damage to the small intestinal lining and malabsorption of nutrients. Celiac disease is activated in genetically susceptible individuals by dietary exposure to gluten in wheat and gluten-like proteins in rye and barley. Symptoms are diverse and include gastrointestinal and extraintestinal manifestations. Treatment requires strict adherence to a gluten-free diet. The Internet is a major source of health information about celiac disease. Nonetheless, information about celiac disease that is available on various websites often is questioned by patients and other health care professionals regarding its reliability and content.

**Objectives:**

To determine the accuracy, comprehensiveness, transparency, and readability of information on 100 of the most widely accessed websites that provide information on celiac disease.

**Methods:**

Using the search term *celiac disease*, we analyzed 100 of the top English-language websites published by academic, commercial, nonprofit, and other professional (nonacademic) sources for accuracy, comprehensiveness, transparency, and reading grade level. Each site was assessed independently by 3 reviewers. Website accuracy and comprehensiveness were probed independently using a set of objective core information about celiac disease. We used 19 general criteria to assess website transparency. Website readability was determined by the Flesch-Kincaid reading grade level. Results for each parameter were analyzed independently. In addition, we weighted and combined parameters to generate an overall score, termed website quality.

**Results:**

We included 98 websites in the final analysis. Of these, 47 (48%) provided specific information about celiac disease that was less than 95% accurate (ie, the predetermined cut-off considered a minimum acceptable level of accuracy). Independent of whether the information posted was accurate, 51 of 98 (52%) websites contained less than 50% of the core celiac disease information that was considered important for inclusion on websites that provide general information about celiac disease. Academic websites were significantly less transparent (*P* = .005) than commercial websites in attributing authorship, timeliness of information, sources of information, and other important disclosures. The type of website publisher did not predict website accuracy, comprehensiveness, or overall website quality. Only 4 of 98 (4%) websites achieved an overall quality score of 80 or above, which a priori was set as the minimum score for a website to be judged trustworthy and reliable.

**Conclusions:**

The information on many websites addressing celiac disease was not sufficiently accurate, comprehensive, and transparent, or presented at an appropriate reading grade level, to be considered sufficiently trustworthy and reliable for patients, health care providers, celiac disease support groups, and the general public. This has the potential to adversely affect decision making about important aspects of celiac disease, including its appropriate and proper diagnosis, treatment, and management.

## Introduction

The Internet is a major source of health information [1]. Most Internet users in the United States have searched for health information online [2]. Studies of current Internet usage found that over 93% of patients with varying digestive diseases may seek Web-based health information [3-5]. Since many individuals rely on websites as a major source of information about celiac disease, the gluten-free diet, and gluten intolerance (also termed gluten sensitivity), it is essential that the information available to those individuals on the Web be of high quality.

Celiac disease is an autoimmune digestive disease that is activated in genetically susceptible individuals by dietary exposure to wheat gluten and similar proteins, termed secalins and hordeins, in rye and barley [6,7]. Celiac disease is estimated to occur in approximately 1% of the US population [7,8]. However, as many as 95% of the estimated 2.75 million Americans with celiac disease have not had it diagnosed and, in those with a diagnosis, the average delay to diagnosis is estimated to be from 4 to 11 years [8-10]. Awareness among health care professionals of the highly variable presentations of celiac disease remains low, and many are not aware of the diversity of presenting gastrointestinal and extraintestinal manifestations that can include, for example, abdominal pain, diarrhea or constipation, fatigue, iron deficiency anemia, premature-onset osteoporosis, depression, irritability, and neuropathy [6,7,10,11]. Based on its protean symptoms, it is not surprising that patients who suspect their symptoms may be caused by celiac disease often search for health information on the Web, self-diagnose, and treat their symptoms with a gluten-free diet [12]. Those who have a definite or presumptive diagnosis of celiac disease [6] also are likely to use the Internet to seek information about this disease, especially its treatment, which requires a strict dietary change but does not require prescription pharmaceuticals. Self-diagnosis and -treatment of celiac disease can result in misdiagnosis or a delay in diagnosis of other diseases and disorders, and renders the future diagnosis of celiac disease significantly more difficult after the individual has initiated a gluten-free diet [6].

Several studies have expressed concern about inaccurate health information on the Internet [13-19]. Individuals who rely on the Internet to obtain health information may be using material that is not evidence based, or does not meet standards of care, to self-diagnose and self-treat health conditions [2,20]. In addition, several reports describe physical and emotional harm resulting from misinformation on the Internet, and such instances are estimated to be underreported [17,18]. Additionally, studies examining perceptions of online Web-based health information indicate that many physicians feel such information is not accurate and has the potential to be detrimental to patient health outcomes [16,19].

Recently, there has been a marked proliferation of websites providing information about celiac disease. These websites typically are published by academic medical centers, commercial organizations, nonprofit organizations (eg, celiac support groups or governmental agencies), or other professionals (nonacademic). However, there has been little to no analysis of which sites provide accurate and reliable information. Such information is essential for patients, support groups, and health care providers to make reasonable, informed decisions and provide appropriate advice regarding the diagnosis and treatment of celiac disease. Only one study, published in 2004, investigated websites providing information about celiac disease. The study found that 66% of websites scored less than 50% for overall accuracy, and 15.9% of websites contained inaccurate information that was potentially harmful [21]. In addition, almost two-thirds of the 63 websites examined (ie, all the celiac disease websites available for analysis at that time) scored less than 50% for overall transparency, failing to provide sufficient information about such variables as author credentials, information sources, creation or revision dates, and funding sources.

The aim of this study was to (1) determine the accuracy, comprehensiveness, transparency, and reading grade level of the most frequently accessed websites that provide information about celiac disease, (2) develop an overall quality score for those websites, and (3) investigate which variables and types of website publishers might predict website accuracy and overall quality.

## Methods

### Website Selection

We selected websites in November 2010 using Google, Microsoft Bing, and Yahoo! search engines on the World Wide Web and the search term *celiac disease*. Websites were considered for inclusion if they were targeted at consumers and provided information about celiac disease in the English language. Websites were excluded that contained broken links, required an access fee, or consisted only of links, gluten-free recipes, blogs, videos, or non-US-based local information.

The Google, Bing, and Yahoo! search engines accounted for 92.1% of all Internet searches in 2010 [22]. According to the Nielsen Company, 65.1% of searches are made through the Google search engine, 13.9% via Microsoft’s Bing, and 13.1% through Yahoo! [22]. For this study, we first selected the top 100 websites from each of those search engines, yielding a total of 300 websites. Websites were given a weighted score based on the search engine’s share of searches (65.1%, 13.9%, and 13.1%, for Google, Bing, and Yahoo!, respectively) and the website’s rank within each search engine (1–100). For initial analysis, we selected the top 100 sites with the highest weighted scores that met the inclusion criteria. On retest 2 months later, 2 of the 100 websites initially selected contained broken links and were excluded from the study. Final analysis included the remaining 98 websites.

Search engines use algorithms to conduct searches, which are personalized to past searches, geographic location, popularity, and quality of content, among other variables. Thus, it is possible for each individual searching the Web to obtain different results when using modern search engines. This can lead to search engine bias [23]. To address this, we disabled customizations based on search activity in Google. To our knowledge, there is no known method of disabling customizations in Yahoo! or Bing. Since cookies store data from past searches, they were also disabled, so data collected from past searches would not influence future searches. We included Web pages linked to each of the websites selected for analysis in that website’s analysis if the primary URL of the linked webpage was the same as the URL of the selected website (eg, www.csaceliacs.org; www.csaceliacs.org/Celiac Disease.php).

The 100 websites initially chosen for analysis fell into 1 of 4 categories: academic, commercial, nonprofit, or professional (nonacademic). Academic Websites were those posted by university-based or -affiliated medical institutions; commercial websites were those posted by for-profit companies; nonprofit websites were posted by not-for-profit organizations or federal, state, or local government agencies; and professional (nonacademic) websites were posted by various types of professionals not associated with academic medical centers.

### Criteria Used to Assess Website Accuracy

Website accuracy was probed and scored by determining the accuracy of information each website provided that addressed a core information base for celiac disease (see Multimedia Appendix 1). We developed the core information base for celiac disease by culling what we considered minimum essential information about celiac disease from a much broader information database on celiac disease, developed at the Warren Medical Research Center for Celiac Disease and based on the best available evidence. What constituted essential core information about celiac disease was selected following a review by a panel of 5 celiac disease experts at the University of California, San Diego (3 gastroenterologists and 2 registered dieticians) and input from patients in San Diego celiac disease support groups . The core information base encompassed information related to the definition, etiology, prevalence, genetics, symptoms, diagnosis, treatment, and complications of celiac disease. Accuracy of disease-specific information in the core provided by the 98 websites was measured as a dichotomous variable. Each piece of disease-specific information provided by the website that was part of the core body of information (Multimedia Appendix 1) was scored 1 for accurate or 0 for inaccurate. Accuracy scores were calculated as a fraction, where the denominator equaled the number of pieces of core information that were present and the numerator equaled those pieces of core information that were both present and correct. This resulted in scores between 0 and 1, with more accurate sites scoring closer to 1 and less accurate sites scoring closer to 0. This scoring algorithm did not penalize accuracy for a lack of comprehensiveness in terms of the amount of information provided by each website. We report data as a score for accuracy of the 98 websites and accuracy for the websites according to the type of website publisher. We further report the percentage of websites from each type of website publisher that scored 95% or greater for accuracy. This level of accuracy was a priori considered a minimum for providing patients, health care providers, and the public a reasonably high level of confidence that the information posted was accurate, irrespective and independent of the amount or diversity of celiac disease information provided by the website.

### Comprehensiveness of Disease-Specific Information

Comprehensiveness is a measure of how much of the core information base about celiac disease (Multimedia Appendix 1) was provided by the website. Comprehensiveness was probed and scored cumulatively, with a score of 1 for information provided and 0 for information not provided, with a maximum possible score for each website of 70. Scores for comprehensiveness were based on whether information was provided with respect to the core information base for celiac disease, irrespective of whether the information provided was accurate.

### Website Transparency

Transparency was probed and scored based on information provided on the website relevant to characteristics that included disclosure of authorship, attribution of sources, whether the information was current (ie, dates of website creation and updating), and the presence of publisher disclosures, using a 5-point Likert scale (Multimedia Appendix 2). We adapted the 19 criteria used to assess transparency from those used by others to assess general health information on the Internet [21,24-29].

### Website Reading Grade

The ability of individuals to have an opportunity to understand health information on the Web relies on their ability to easily read the information [14,30-32]. We determined the US grade level of the text in each website using the Flesch-Kincaid reading grade level [33] and an online reading grade calculator [34].

### Website PageRank

PageRank is a Google rating system for websites that is based on the number and quality of backlinks (ie, links pointing to a given website) [35]. Websites were ranked on a scale of 0 to 10. We collected PageRank values for each website to determine whether websites with a higher PageRank also had higher scores for accuracy, comprehensiveness, and transparency, or lower reading grade levels.

### Website Quality Score

We determined an overall score termed website quality by cumulatively assessing the combined features of accuracy, comprehensiveness, transparency, and reading grade level, but not PageRank, for each of the 98 websites and for websites according to the type of website publisher. Each parameter contained in the quality score was multiplied by a relative weighting based on its a priori perceived importance. Accuracy was considered the single most important component of website quality, with comprehensiveness, transparency, and reading grade level being important, but to a lesser degree. The relative weightings given each parameter for accuracy, comprehensiveness, transparency, and reading grade level as components of the quality score were 10, 5, 4, and 4, respectively. Quality scores ranged from 0 to 10. We also determined a priori that, to be reliable and of reasonable quality, a website should obtain a minimum quality score of 8.0.

### Website Reviewers

We recruited and trained 3 reviewers, independent from the authors responsible for study concept and design and having no prior association with the Warren Medical Research Center for Celiac Disease and no prior familiarity with any of the websites, to score the websites for accuracy, comprehensiveness, transparency, reading grade level, and PageRank. Each reviewer independently scored each of the initial 100 websites. As a test of interrater reliability, we compared the scoring of disease-specific accuracy, comprehensiveness, and transparency by each of the 3 reviewers. Intrarater reliability was determined by having each reviewer rescore 10 websites 2 months after their initial scoring. Since information on the Internet changes frequently, website reviews were completed within 8 weeks of the initial search for celiac disease.

### Statistical Analysis

Mean scores of the websites, based on accuracy, comprehensiveness, transparency, reading grade level, PageRank and quality, according to the type of website publisher, were compared using analysis of variance and post hoc pairwise *t* tests with Holm adjustment for multiple comparisons [36]. The independence of website type and the proportion of websites with accuracy greater than 90% and 95% were assessed with Pearson’s chi-square tests, followed by pairwise Holm-adjusted Pearson chi-square tests. We used box-and-whisker plots to show data dispersion and skewness [37,38]. Interquartile range was calculated as the difference between the third and first quartiles (interquartile range = Q_3_ - Q_1_).

We used the Pearson product–moment correlation coefficient to assess the linear association between accuracy, comprehensiveness, transparency, reading grade level, and PageRank, and correlations stratified by type of website publisher. A locally weighted scatter plot smoothing was used to assess nonlinear trends [39].

Intrarater reliability was measured with the test–retest method. Test–retest reliability was assessed using the Pearson correlation coefficient. Interrater reliability among the 3 reviewers was determined using the intraclass correlation coefficient for agreement [40].

## Results

Of the 100 websites selected for initial analysis, 47 were present in the top 100 websites from Google, Bing, and Yahoo!, whereas 15 were common to Bing and Yahoo! and 38 were present only in Google’s top 100 websites. We excluded 2 websites after initial analysis since they contained broken links when reanalyzed 2 months later. Of the 98 websites whose data we included in the final analysis, 11 were from academic medical centers, 48 were from commercial publishers, 28 were from nonprofit organizations, and 11 were from professionals not affiliated with academic medical institutions.

### Overall Website Scores

Overall accuracy scores for celiac disease core information on the 98 websites ranged from 0.62 to 1.00 (mean 0.93, SD 0.07; median 0.95; maximum obtainable score 1.0). Comprehensiveness scores ranged from 6.3 to 61.3 (mean 34.8, SD 12.5; median 32.7; maximum obtainable score 70), and transparency scores ranged from 0.27 to 0.80 (mean 0.52, SD 0.12; median 0.52; maximum obtainable score 1.0).

### Website Scores by Type of Website Publisher

**Figure 1:**
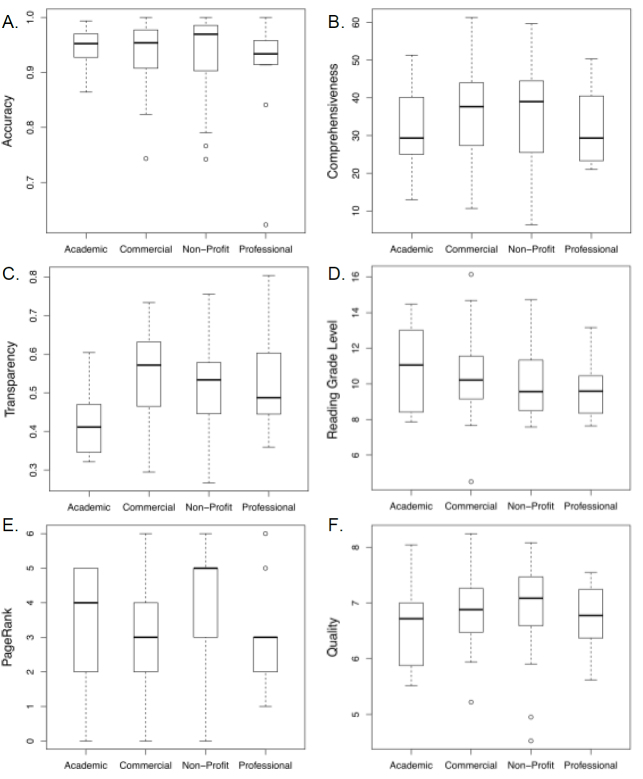
Distribution of accuracy (A), comprehensiveness (B), transparency (C), reading grade level (D), PageRank (E), and quality scores (F) for the studied websites. The bottom and top of the box-and-whisker plots represent the 25th and 75th percentiles (ie, lower and upper quartiles, respectively). Crossbar is the median (50th percentile). Ends of whiskers represent data within 1.5 times the interquartile range of the lower and upper quartiles. Data not included between whiskers are shown as outliers (small circle). Spacing between the parts of the box indicates degree of dispersion and skewness of the data. Panel A, *F*_3,94_ = 0.70; Panel B, *F*_3,94_ = 0.34; Panel C, *F*_3,94_ = 4.07; panel D, *F*_3,90_ = 1.00; panel E, *F*_3,87_ = 2.71; panel F, *F*_3,94_ = 0.91.

Our study design ranked the most important attribute of a website as the accuracy of its posted information, irrespective of the quantity of information provided. We had posited that one might be able to predict website accuracy based on the type of website publisher. Therefore, we compared website accuracy according to the type of website publisher (ie, academic, commercial, nonprofit, and other nonacademic professional). Mean (Table 1) and median (Figure 1, panel A) scores for accuracy did not differ significantly according to the type of website publisher. Our study design a priori considered that, to qualify as a practically useful resource for the education of patients, health care providers, and the public, a minimum of 95% or more of the celiac disease-specific information provided by a website should be accurate, independent of how much information was provided (ie, independent of comprehensiveness). Overall this level of accuracy was obtained by 51 of 98 (52%) of the websites. Of note, the information presented by 7 of 11 (64%) academic websites met this criterion, whereas the information on 3 of 11 (27%) professional (nonacademic) websites was 95% or more accurate (Table 1), although each type of website had a wide range of scores for accuracy (Figure 1, panel A).

**Table 1:** Website scores for accuracy, comprehensiveness, transparency, reading grade, PageRank, and quality by website type.

Criterion	Academic (n = 11)^a^	Commercial (n = 48)^a^	Nonprofit (n = 28)^a^	Professional (n = 11)^a^	Combined (n = 98)^a^	*P* value
Accuracy, mean (SD)	0.95 (0.04)	0.94 (0.06)	0.94 (0.07)	0.91 (0.10)	0.93 (0.07)	.56^b^
Accuracy >0.95, n (%)^c^	7 (64%)	26 (52%)	15 (57%)	3 (27%)	51 (52%)	.31^d^
Accuracy >0.90, n (%)	10 (91%)	39 (80%)	22 (81%)	9 (82%)	80 (82%)	.84^d^
Comprehensiveness, mean (SD)	32.4 (12.1)	35.5 (12.3)	35.4 (13.9)	32.4 (11.4)	34.8 (12.5)	.80^b^
Transparency, mean (SD)	0.42 (0.09)	0.55 (0.11)	0.52 (0.11)	0.53 (0.13)	0.52 (0.12)	.01^b^
Reading grade level, mean (SD)	10.9 (2.6)	10.4 (2.2)	10.0 (2.0)	9.6 (1.6)	10.3 (2.1)	.40^b^
PageRank, mean (SD)	3.6 (1.7)	3.1 (1.7)	4.2 (1.5)	3.0 (1.4)	3.4 (1.7)	.05^b^
Quality, mean (SD)	6.5 (0.79)	6.9 (0.61)	6.9 (0.83)	6.7 (0.65)	6.8 (0.70)	.44^b^

^a^ Number of websites analyzed.

^b^ *P* values from analysis of variance.

^c^ Number of websites (% of websites).

^d^ *P* values from Pearson chi-square test.

Comprehensiveness is a measure of the amount of important core information on celiac disease provided by the websites, irrespective of its accuracy. Academic, commercial, nonprofit, and nonacademic professional sites each had a broad range of scores for comprehensiveness (Table 1 and Figure 1, panel B). Of 98 websites, 51 (52%) scored less than 35 for comprehensiveness, indicating they provided less than 50% of the core celiac disease information that was considered important for inclusion on websites that provide general information on celiac disease. Comprehensiveness scores did not differ significantly according to the type of website publisher (Table 1 and Figure 1, panel B).

Transparency and PageRank significantly differed according to the type of website publisher (Table 1 and Figure 1, panels C and E). Post hoc pairwise comparisons revealed that academic websites were significantly less transparent than commercial websites (*P* = .005) (Figure 1, panel C), and websites published by nonprofit organizations had a higher PageRank than commercial websites (*P* = .05) (Figure 1, panel E).

The average US reading level for adults has been reported to be between the 8th and 9th grade [41]. The reading grade levels of the websites ranged from grade 4.5 to 16.2 (ie, 4-year college graduate) with a median reading grade of 9.8. Reading grade did not differ significantly among academic, commercial, nonprofit, and professional sites (Table 1 and Figure 1, panel D).

**Figure 2:**
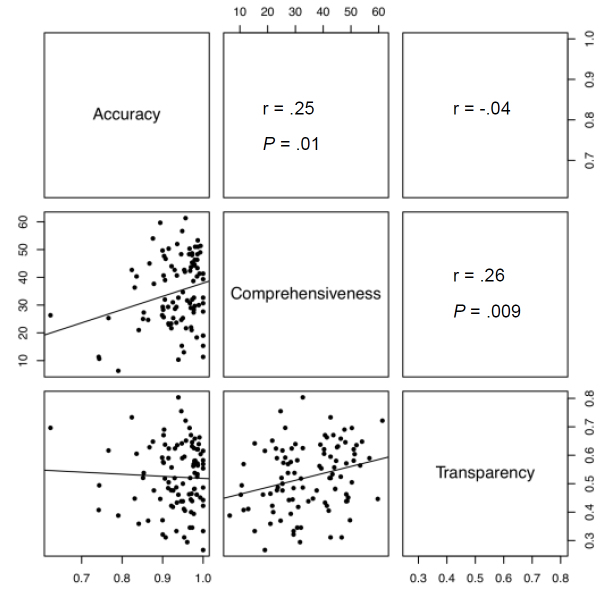
Matrix of scatter plots of accuracy, comprehensiveness, and transparency of the websites. The correlation coefficients (*r*) and significance values (*P*) are shown within the boxes. There was a significant positive correlation between website accuracy and comprehensiveness (*r* = .25, *P* = .01) (dot plot shown in left middle panel) and between website comprehensiveness and transparency (*r* = .26, *P* = .01) (dot plot shown in bottom middle panel). There was no significant correlation between accuracy and transparency (*r* = -.04) (dot plot shown in left bottom panel).

Analysis of correlations between accuracy, comprehensiveness, transparency, reading grade level, and PageRank revealed a significant positive linear correlation between website accuracy and website comprehensiveness (*r* = .25, *P* = .01) and between website comprehensiveness and website transparency (*r* = .26, *P* = .009) (Figure 2). In contrast, we found a significant negative correlation between accuracy and the inclusion of personal testimonies on websites (*r* = –.24, *P* = .02).

### Website Quality

Website quality is a weighted cumulative average of website accuracy, comprehensiveness, transparency, and reading grade level (Table 1 and Figure 1, panel F). A priori our study design had set a quality score of 8.0 or greater as a minimum acceptable score for a website to be regarded as reliable and of reasonable quality. Actual data analysis revealed that this cut-off produced websites that mostly ranked in or very near to the top quartile for accuracy, comprehensiveness, and transparency and had an optimal, reasonable reading grade level. Nonetheless, only 4 of the 98 websites included in our final analysis had a quality score of 8.0 or greater. Of the 4 websites, 2 were from nonprofit publishers (1 celiac support group and 1 government source), 1 was from an academic institution, and 1 was from a commercial publisher (Table 2).

**Table 2:** URLs, website type, accuracy, comprehensiveness, transparency, reading grade level, PageRank, and quality scores of websites with quality scores of 8.0 or higher.

URL	Website type	Accuracy	Comprehensiveness	Transparency	Reading	PageRank	Quality
1^a^	Nonprofit	0.99	53.3	0.56	7.6	6	8.1
2^b^	Nonprofit	0.99	51.0	0.62	7.9	6	8.1
3^c^	Academic	0.99	51.3	0.60	8.2	5	8.0
4^d^	Commercial	0.96	61.3	0.72	10.2	6	8.2

^a^ http://www.celiac.org. Archived at http://www.webcitation.org/63IJ5QRRw.

^b^ http://www.digestive.niddk.nih.gov/ddiseases/pubs/celiac. Archived at http://www.webcitation.org/63FT3dm7I.

^c^ http://celiaccenter.ucsd.edu. Archived at http://www.webcitation.org/63FT9XBSa.

^d^ http://celiacdisease.about.com. Archived at http://www.webcitation.org/63FTCJQij.

**Figure 3:**
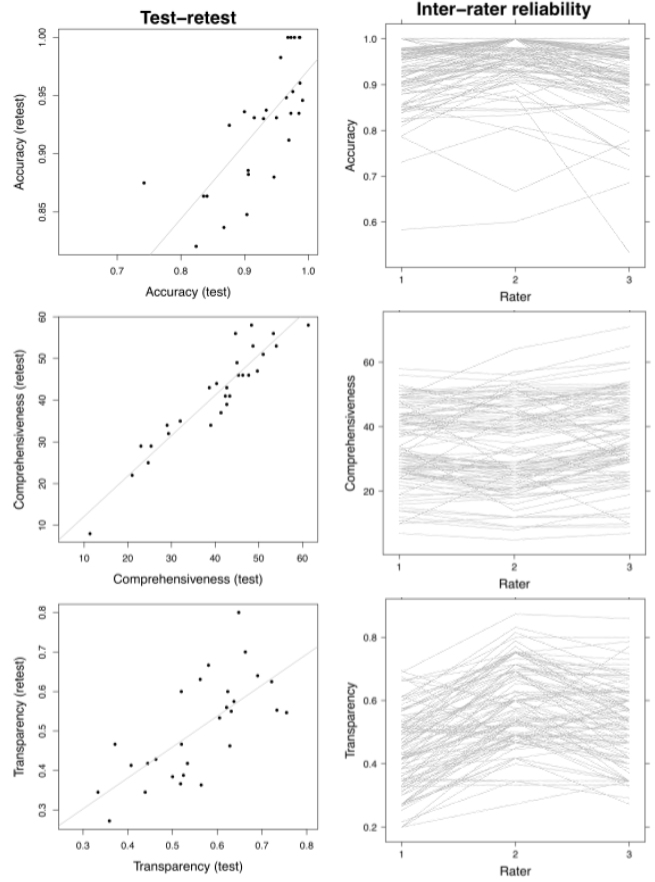
Scatter plots of test-retest scores (left panels), and line plots of interrater reliability (right panels). Each dot on the left set of panels shows the test score (horizontal axis) and retest score (vertical axis) for a single website. For the right set of panels, each website is represented by a single gray line. There is a statistically significant positive correlation between the test and retest scores for accuracy (*r* = .75, 95% confidence interval [CI] .52-.88), comprehensiveness (*r* = 0.94, 95% CI .87-.97), and transparency (*r* = .79, 95% CI .59-.90). Interrater reliability statistics show statistically significant intraclass correlation coefficients among the 3 reviewers for the scoring of accuracy (*r* = .68, 95% CI .6-.8), comprehensiveness (*r* = .85, 95% CI .8-.9), and transparency (*r* = .61, 95% CI .5-.7).

### Intrarater and Interrater Reliability

Intrarater reliability revealed a statistically significant positive correlation between the test and retest scores for accuracy (*r* = .75, 95% confidence interval [CI] .52–.88), comprehensiveness (*r* = .94, 95% CI .87–.97), and transparency (*r* = .79, 95% CI .59–.90). Interrater reliability statistics (intraclass correlation coefficient) showed almost perfect agreement among the 3 reviewers for the scoring of comprehensiveness (*r* = .85, 95% CI .8–.9), and moderate to strong agreement for the scoring of accuracy (*r* = .68, 95% CI .6–.8) and transparency (*r* = .61, 95% CI .5–.7) (Figure 3).

## Discussion

Individuals with celiac disease or those seeking information about celiac disease often turn to websites for that information. Moreover, gastroenterologists, other health care professionals, and celiac support groups who counsel celiac disease patients frequently refer patients to the Internet as a trusted source of information. Websites can be a major source of information on the etiology, genetics, and pathogenesis of celiac disease, as well as its diagnosis, treatment, management, and complications. Therefore, it is essential that website information on celiac disease be accurate, sufficiently comprehensive, transparent, and at an appropriate reading grade level.

### Accuracy

We used a set of core information about celiac disease to probe and analyze the accuracy of the information on each of 98 websites. We considered the accuracy of the information about celiac disease on a website to be its single most important characteristic, irrespective of how much information a particular website provided. Conveying information that is not accurate can adversely affect the consumer’s understanding of this disease, and more importantly its diagnosis, treatment, and understanding of potential complications, depending on the nature of the misinformation being promulgated. An early study using the same search term as used herein [21] did not distinguish accuracy from comprehensiveness and penalized sites for lack of accuracy based on absent information. Our analysis of accuracy independent of comprehensiveness is a significant strength of the present study.

Based on a diverse range of symptoms and presentations, patients frequently self-diagnose celiac disease. Moreover, given that celiac disease is treated by diet rather than prescription pharmaceuticals, patients can easily decide to self-treat and commit themselves to a gluten-free diet based, at least in part, on inaccurate or incomplete information obtained from surfing the Web. Self-diagnosis can result in misdiagnosis, a delay in the diagnosis of other underlying disorders, and inappropriate or suboptimal treatment. In addition, self-treatment with a gluten-free diet renders it difficult for physicians to subsequently make a correct diagnosis by serology and a small intestinal mucosal biopsy, especially if patients are not willing to undergo a diagnostic gluten challenge, which frequently is the case.

To provide assurance that the information the consumer relies on is mostly correct, a panel of celiac disease experts decided a priori that a website’s information about celiac disease should be at least 95% accurate. This was an arbitrary, but nonetheless justifiable, cut-off. Overall, 52% of the websites analyzed achieved 95% or greater accuracy. Nonetheless, it is disappointing that the information content on approximately 20% of commercial, nonprofit, and professional websites was less than 90% accurate. Inaccuracies across the websites were most common for (1) the definition of celiac disease (eg, incorrectly calling celiac disease a gluten allergy), (2) the prevalence of disease (eg, errors in prevalence of celiac disease, its occurrence among different racial or ethnic groups, stating that celiac disease is rare), (3) the proper diagnosis of celiac disease in adults and children (eg, use of blood tests or small-bowel mucosal biopsy), and (4) various aspects of the gluten-free diet (eg, use of oats in the diet).

### Comprehensiveness

We used comprehensiveness as a measure of the quantity of the core information base on celiac disease that each website provided. Overall, less than 50% of the websites provided at least 50% of the core information that we considered important for inclusion on a general site about celiac disease. We found no correlation between website deficiencies in comprehensiveness and the type of website publisher.

### Transparency

Transparency measures the extent to which a website discloses relevant general information such as the website author’s credentials, the website’s sources of information, dates of website creation and revision, and website funding sources. Whereas commercial, nonprofit, and professional website publishers on average provided at least 50% of the information being sought in that regard, academic sites did not. Although ranking highest in information accuracy, academic sites surprisingly were significantly less transparent than commercial websites. We note that the DISCERN instrument, which is often used to assess website quality, primarily assesses transparency regarding treatment, and does not assess website accuracy [42,43].

### Readability

The reading grade level is the grade level of education an individual in the United States would need to achieve to easily read the information provided by the website. Documents written at the mid 8th to 9th grade level or lower are suitable for reading by the average adult US population according to the Department of Health and Human Services [41]. Only 50% of websites we analyzed met that criterion. Although we did not find statistically significant differences in the reading grade level among different types of website publishers, we did find a wide range of reading grade levels among websites that ranged from 4th grade to college graduate level. If an individual has difficulty reading the website material, there is a greater potential for the information to be misunderstood and misapplied. Although several scoring systems can be used to assess readability, we selected the one most widely used and suited to this study. Nonetheless, we recognize that none of the instruments available are specifically tailored to the readability of medical information by the general public or tailored to specifically analyze the reading grade level of celiac disease-specific information. Further, individuals with a high school diploma may still read at an 8th grade level, and reading grade level does not equate to the capacity to understand health information.

### PageRank

The median PageRank for all sites for which data were available was 3 on a scale of 0 to 10, with 10 being highest. PageRank is a link-analysis algorithm [35] used by the Google Internet search engine . The algorithm assigns a numerical weighting to each element of a hyperlinked set of documents, with the purpose of assessing its relative importance within the set. The lowest median PageRanks were those of professional (nonacademic) and commercial sites, with the highest being nonprofit sites, whose median PageRank was significantly greater than that of commercial sites. A possible explanation for this result may be the greater number of backlinks on nonprofit sites, which include government sites and celiac support groups that may naturally obtain more backlinks simply because they may be seen by other websites as more reliable sources of information.

### Quality Score

Individuals surfing the Web seeking information usually want that information quickly. They often do not have the resources or time to analyze each website for its quality (ie, as defined herein, a weighted composite of accuracy, comprehensiveness, transparency, and reading grade level). Moreover, individuals search for information on celiac disease for different reasons and might, for example, be seeking information on disease epidemiology, pathogenesis, or even more likely diagnosis, treatment, and complications. In each instance, the accuracy of the information is very important. However, to be most useful, the information presented also should be sufficiently comprehensive and readable, the sources and timeliness of the information should be indicated, and disclosures regarding authorship and funding sources should be provided. To integrate the various parameters analyzed in this study, we developed an overall rating of the websites, which we termed the quality score. We based this score, which ranges from 0 to 10, on an arbitrary weighted average of each of the key parameters we analyzed, based on the perceived relative importance of those parameters. The median quality score of all the sites combined was 6.8, and we found no significant difference in quality scores among academic, commercial, nonprofit, and professional sites. Overall, the type of website publisher did not predict the level of quality of an individual website. However, within each type of website publisher, individual website quality varied markedly.

### Correlations

We tested for correlations among the parameters of accuracy, comprehensiveness, transparency, reading grade level, or PageRank for the websites to determine whether any one parameter might predict the results obtained for any of the other parameters. Website accuracy for individual websites significantly correlated with website comprehensiveness, indicating that websites with the highest accuracy also tend to be those that provide the greatest amount of celiac disease-specific information. In addition, the websites that provided the most information also tended to be those with the greatest transparency. Conversely, websites that contained personal testimonials tended to be less accurate as indicated by a significant negative correlation between those parameters.

### Study Design

Several features of the design of this study contribute to its strength. First, the sample size and the method of selection we used to choose the final 98 websites for analysis were important. Among other variables, search engines use algorithms to conduct searches that are personalized to past searches, popularity, and quality of the content. We circumvented this in part by disabling customizations on Google and cookies on all 3 search engines. Since each person still may obtain a slightly different search, we used a large sample size to include the websites that the majority of individuals would obtain in their search results. By obtaining 100 initial websites from the each of the 3 search engines and weighting those websites for their frequency of use by the general population in the United States, we achieved a large sample size of the sites most often viewed by individuals searching for information using the search term *celiac disease* and concurrently minimized the standard error of reliability [44-46].

An additional strength of this study was the use of 3 website raters. The raters were trained to review the sites but were not otherwise involved with the study design or the Wm. K. Warren Medical Research Center for Celiac Disease. Each rater independently analyzed each of the 100 websites using objective, easily scored criteria. We achieved very strong positive correlations between the test and retest scores of the individual reviewers for comprehensiveness, and moderately strong positive correlations for accuracy and transparency; and almost perfect interrater agreement for comprehensiveness, with moderate to strong levels of agreement for accuracy and transparency.

### Limitations

We analyzed the top 100 consumer-accessed websites from the major search engines, which provided a meaningful number of sites for analysis. This sample included most of the academic medical centers in the US that have centers of excellence in celiac disease. However, our sample of websites did not have an equal representation of academic, commercial, nonprofit, and professional (nonacademic) website publishers. Balancing the number of websites from each type of website publisher would require specific website selection based on publisher type and would not achieve our aim of analyzing the most highly accessed websites.

The study design used the phrase *celiac disease* as the search term. We determined from patient groups and health care professionals that this was the search term most likely to be used by individuals seeking general information about celiac disease. However, we recognize not all individuals might use that term. Thus, others searching for information about celiac disease might use focused search phrases such as *celiac disease symptoms*, *celiac disease diagnosis*, or *celiac disease treatment* or, alternatively, general terms such as *gluten free, gluten intolerant*, or *gluten sensitive*, which may yield a different set of websites.

The core information base for celiac disease we used to grade website accuracy and comprehensiveness was developed by academic professionals’ expert in celiac disease. This core information was a sampling of evidence-based information considered relevant for the purpose of probing the accuracy and content of the websites analyzed. One could discuss why some items of information were or were not included in our core information base. However, the base was of sufficient scope that some additions or deletions are unlikely to have significantly affected the results and conclusions of this study. We did not address how the specific inaccuracies in information on the various websites might adversely affect consumer understanding of this disease, its diagnosis, or treatment, or how various inaccuracies have different levels of potential for causing harm.

Website quality was a composite score based on weightings provided to its 4 component scores, based on the perceived importance of each component to website quality. Although the weighting we used was derived from a consensus of several experts in the field, different experts could arrive at different weightings. Nonetheless, there should be uniform agreement that the relative weighting of accuracy is the most important single weighting for website reliability and reader education and exceeds that of comprehensiveness, since topics not addressed on one website are likely to be covered by another [47].

Website design features and layout may render one website more or less difficult to navigate than another. Although this has implications for the ease of the reader finding information, our study did not evaluate differences in website design features, as such evaluations tend to be relatively subjective. We also did not compare the accuracy, comprehensiveness, transparency, and readability of celiac disease information available on the Web with that presented by other media (eg, television, magazines, or newspapers).

Finally, we recognize that increasing the number of reviewers might further improve interrater reliability statistics [48]. We also note that websites related to this disease are in a continuous state of change.

### Conclusions

Websites are a major source of information about celiac disease for patients, the public, and health care providers. However, it is difficult for the average individual seeking information on celiac disease to assess the reliability and overall quality of the material being presented. The 98 most accessed celiac disease websites were shown to be highly variable in the accuracy and comprehensiveness of the data presented, as well as in their transparency, readability, and overall quality. Furthermore, the type of website publisher was not a predictor of individual website accuracy, comprehensiveness, reading grade level, or overall quality. Based on objective criteria and a rigorous review process, only 4 of 98 websites achieved an overall quality score deemed sufficient to judge the information on the website as reasonably trustworthy and reliable. Since the type of website publisher alone is not a valid indicator of accuracy and comprehensiveness of information that focuses on celiac disease, we suggest that all types of website publishers addressing celiac disease pay greater attention to the accuracy, comprehensiveness, transparency, and readability of the information they provide. We further suggest that a regular ongoing review and evaluation of the most highly accessed websites by experts in celiac disease may be helpful to patients, health care professionals, members of celiac support groups, and those in the public seeking accurate and reliable information about celiac disease on the Internet.

## Multimedia Appendix 1

Core information base for celiac disease used to evaluate accuracy and comprehensiveness of websites.

## Multimedia Appendix 2

Criteria used to evaluate website transparency.

## Abbreviations

CI: confidence interval
