# Supplementary material for: Can Consumers Trust Web-Based Information About Celiac Disease? Accuracy, Comprehensiveness, Transparency, and Readability of Information on the Internet
Source: Interact J Med Res. 2012 Apr 4;1(1):e1. doi: 10.2196/ijmr.2010 (PMC3626119; doi:10.2196/ijmr.2010)
Supplement: Supplementary file 1 [file ijmr_v1i1e1_app1.pdf]

Supplementary Table 1. Core information base for celiac disease used to evaluate accuracy and comprehensiveness of websites

Celiac Disease medical information

Definition:
- Inflammatory condition
- Small intestine
- Triggered by gluten ingestion
- Results in villous atrophy/damage of small intestine
- Causes malabsorption of nutrients
- Damage improves or heals when gluten is excluded
- Lifelong disease or no known cure
- Differs from gluten allergy, non-celiac gluten intolerance or sensitivity

Etiology: immune response/T cell response to gluten, autoimmune disease

Prevalence:
- Between 1:100 and 1:200
- Relatively common
- Affects different races and ethnicities

Hereditary/Genetics:
- Requires a genetic predisposition
- Associated with HLA DQ2 & DQ8 or HLA-associated or HLA class II.
- Genes are necessary but not sufficient for celiac disease to occur, or having HLA DQ2 or DQ8 does not mean individual will get celiac disease.

Symptoms:
- May present with gastrointestinal or non-gastrointestinal symptoms
  List of Intestinal symptoms (for comprehensiveness score only)
  List of extra-intestinal symptoms (for comprehensiveness score only)
- Celiac disease is associated with other diseases/conditions
  List of associated conditions (for comprehensiveness score only)
- Celiac disease may be silent without apparent symptoms
  List of associated conditions (for comprehensiveness score only)
- May also occur in overweight or obese individuals

Diagnosis:
- Use blood test to screen. Must include mention of IgA TTG, or IgA EMA or deamidated gliadin peptide (IgA or IgG)
- Confirm by small intestinal biopsy
- A medical diagnosis is necessary prior to starting a gluten-free diet
- Improvement of symptoms on a gluten-free diet
- If GFD already established a gluten challenge may be necessary to confirm
- IgA deficiency may require alternative serologic testing
- High-risk persons should be screened
- Genetic tests alone do not diagnose celiac disease

Treatment:
- Only treatment is gluten-free diet
- Diet must be strict or tiny amounts of gluten may harm the intestine
- Lifelong adherence to gluten-free diet
- Supplementation may be needed to correct nutritional deficiencies

Complications:
- Mention of refractory celiac disease/ enteropathy associated T-cell lymphoma/ early mortality

Supplementary Table 1. (Continued)

Pediatrics

- Breastfeeding may delay or prevent onset of celiac disease
- Blood screen with IgA TTG and IgA EMA is less reliable in children <2 yrs
- Pediatric signs and symptom list (for comprehensiveness score only)

Dermatitis Herpetiformis

- Skin disease with itchy rash and blisters
- Treatment includes gluten-free diet

Gluten-free diet

Where is gluten found?

- Grain sources (i.e. wheat, rye, barley)
- Obvious food sources
- "Hidden" food sources
- Ingredient sources
- Medications and/or Supplements

Safe food sources

- Naturally gluten-free foods
- Gluten-free specialty foods
- Gluten-free grains

Food Labels

- Learning to read food labels
- Food Allergen Law (wheat)
- Wheat free is not gluten free

Oats

- Uncontaminated oats generally safe for most individuals
- Up to 50 grams oats for adults

Cross-contamination at home/ dining out/ or at school

Nutritional management

Follow-up

- Importance of follow-up care
- Monitor nutrient deficiencies

Common nutrient deficiencies

List of nutrient deficiencies (for comprehensiveness score only)

- B-vitamins (thiamine, riboflavin, niacin, folic acid, B6, B12)
- Calcium
- Copper
- Fat-soluble vitamins (A, D, E, K)
- Iron
- Magnesium
- Zinc

Advice to join local celiac support group
