# Supplementary material for: Can Consumers Trust Web-Based Information About Celiac Disease? Accuracy, Comprehensiveness, Transparency, and Readability of Information on the Internet
Source: Interact J Med Res. 2012 Apr 4;1(1):e1. doi: 10.2196/ijmr.2010 (PMC3626119; doi:10.2196/ijmr.2010)
Supplement: Supplementary file 2 [file ijmr_v1i1e1_app2.pdf]

Supplementary Table 2. Criteria used to evaluate website transparency

Authorship

- Disclosure of authorship
- Disclosure of authors' credentials
- Indicates author is a health care professional/expert
- Author's contact details provided

Attribution

- Sources of information are provided?
- References are provided
- Opinion is stated as such
- Working external links provided to scientific reference material/studies?
- Source material is evidence based and peer reviewed?
- Personal testimonies are used
- Personal testimonies are referenced as such
- Contact information is provided for persons whose testimonies are used

Currency

- Date of creation of each page provided
- Date of the last update stated
- The site been updated within the last 6 months

Disclosure

- Declaration of funding source provided?
- Discloses if the site is selling products?
- Discloses if the site carries advertisements?
- Discloses a clear and easy to find privacy policy
